# Superiority of a Novel Mp1p Antigen Detection Enzyme Immunoassay Compared to Standard BACTEC Blood Culture in the Diagnosis of Talaromycosis

**DOI:** 10.1093/cid/ciaa826

**Published:** 2020-06-21

**Authors:** Nguyen T M Thu, Jasper F W Chan, Vo Trieu Ly, Hoa T Ngo, Ha T A Hien, Nguyen P H Lan, Nguyen V V Chau, Jian-Piao Cai, Patrick C Y Woo, Jeremy N Day, Rogier van Doorn, Guy Thwaites, John Perfect, Kwok Yuen, Thuy Le

**Affiliations:** 1 Division of Infectious Diseases and International Health, Duke University School of Medicine, Durham, North Carolina, USA; 2 Oxford University Clinical Research Unit, Ho Chi Minh City, Vietnam; 3 State Key Laboratory of Emerging Infectious Diseases, Carol Yu Centre for Infection, Department of Microbiology, Li Ka Shing Faculty of Medicine, University of Hong Kong, Pokfulam, Hong Kong; 4 Hainan Medical University–University of Hong Kong Joint Laboratory of Tropical Infectious Diseases, Hainan Medical University, Haikou, Hainan, China; 5 Hospital for Tropical Diseases, Ho Chi Minh City, Vietnam; 6 University of Medicine and Pharmacy at Ho Chi Minh City, Ho Chi Minh City, Vietnam; 7 Centre for Tropical Medicine and Global Health, Nuffield Department of Medicine, University of Oxford, Oxford, United Kingdom

**Keywords:** talaromycosis, penicilliosis, *Talaromyces marneffei*, *Penicillium marneffei*, Mp1p enzyme immunoassay

## Abstract

**Background:**

Talaromycosis is an invasive mycosis endemic in Southeast Asia and causes substantial morbidity and mortality in individuals with advanced human immunodeficiency virus (HIV) disease. Current diagnosis relies on isolating *Talaromyces marneffei* in cultures, which takes up to 14 days and is detectable only during late-stage infection, leading to high mortality.

**Methods:**

In this retrospective case-control study, we assessed the accuracy of a novel Mp1p antigen-detecting enzyme immunoassay (EIA) in stored plasma samples of 372 patients who had culture-proven talaromycosis from blood or sterile body fluids (reference standard) and 517 individuals without talaromycosis (338 healthy volunteers; 179 with other infections). All participants were recruited between 2011 and 2017 in Vietnam.

**Results:**

Of cases and controls, 66.1% and 75.4%, respectively, were male; the median age was 33 and 37, respectively. All cases were HIV infected; median CD4 count was 10 cells/μL. At an optical density cutoff of 0.5, the specificity was 98.1% (95% CI, 96.3%–99.0%); the sensitivity was superior to blood culture (86.3% [95% CI, 82.3%–89.5%] vs 72.8% [95% CI, 68.0%–77.2%]) (*P *< .001, McNemar test). The time to diagnosis was 6 hours vs 6.6 ± 3.0 days for blood culture. Paired plasma and urine testing in the same patients (n = 269) significantly increased sensitivity compared to testing plasma alone or testing urine alone (*P *< .001 and *P* = .02, respectively, McNemar test).

**Conclusions:**

The Mp1p EIA is highly specific and is superior in sensitivity and time to diagnosis compared to blood culture for the diagnosis of talaromycosis. Paired plasma and urine testing further increases sensitivity, introducing a new tool for rapid diagnosis, enabling early treatment and potentially reducing mortality.

## Teaser

The novel Mp1p enzyme immunoassay is superior in sensitivity compared to standard BACTEC blood culture and substantially shortens time to diagnosis of talaromycosis. This is a novel non-culture-based tool for rapid diagnosis, enabling early treatment and potentially reducing mortality.

Talaromycosis (formerly penicilliosis) is an invasive mycosis caused by the dimorphic fungus *Talaromyces marneffei* (Tm), which is endemic in Southeast Asia and southern China [1]. Talaromycosis ranks as the third most common human immunodeficiency virus (HIV)–associated opportunistic infection, accounting for up to 16% of HIV admissions, and is a leading cause of HIV-associated death in the highly endemic countries of Thailand, Vietnam, and China [2–5]. Incidence is rising in non-HIV-infected individuals who have a primary or secondary immunodeficiency condition [6] and is rising in immigrants and returning travelers from Southeast Asia [7, 8]. Patients with advanced HIV disease (CD4 count <100 cells/μL) develop an indolent infection over months before progressing to a multiorgan disseminated infection involving the lung, skin, oropharyngeal mucosa, gastrointestinal tract, lymphatic system, liver, spleen, bloodstream, and bone marrow [1, 2]. The mortality despite antifungal therapy is up to 50% at 3 months in both people living with HIV (PLWH) and non-HIV-infected individuals [2, 5, 9, 10].

A critical barrier to reducing talaromycosis mortality is our inability to make an early diagnosis. The current diagnosis relies on culture isolation of Tm from blood and clinical specimens, which takes up to 14 days [2]. In a recent talaromycosis treatment trial in Vietnam, 38 of 573 (6.6%) patients died before culture became positive [9]. Blood culture is positive only when infection progresses to its advanced stage and misses 30% of infections in PLWH and 50% of non-HIV-infected patients [2, 10]. In a cohort of 668 patients in Guangzhou, China, the mortality increased from 24.3% to 50.6% due to late diagnosis, and was 100% when the diagnosis was missed [3].

Antigen detection is accurate, rapid, and inexpensive, does not require sophisticated equipment, and has become a standard diagnostic for other fungal infections including cryptococcosis, aspergillosis, and histoplasmosis [11–13]. Efforts to develop enzyme immunoassays (EIAs) for talaromycosis have been hampered by the use of polyclonal antibodies (PAbs), which lack sensitivity and specificity [14–19]. Recently a promising monoclonal antibody (MAb)–based (MAb 4D1) inhibitory EIA and its immunochromatographic platform were developed in Thailand, but diagnostic accuracy has only been evaluated in small selected clinical samples [20, 21]. We have previously discovered a Tm-unique *MP1* gene, which encodes a galactomannoprotein Mp1p located throughout the cell wall of Tm yeast [22]. The Mp1p antigen is abundantly secreted and is an important virulence factor for Tm [23], making it an ideal and specific target for immunodiagnostics. We have cloned Mp1p and developed an Mp1p EIA using mouse MAbs and rabbit PAbs generated against the recombinant Mp1p [24, 25]. No cross-reactivity occurred with 11 common pathogenic fungi including *Cryptococcus*, *Candida*, *Aspergillus*, and *Histoplasma* species. The Mp1p EIA was positive in 15 of 20 (75.0%) of culture-confirmed talaromycosis patients and negative in 537 of 540 (99.4%) of control participants (15 with invasive mycoses, 525 healthy volunteers), demonstrating excellent analytical and clinical specificities [25]. Here, we report the results of a diagnostic accuracy study of the Mp1p EIA compared with blood culture for the diagnosis of talaromycosis in large patient cohorts in Vietnam.

## MATERIALS AND METHODS

### Ethics Statement

The study was approved by the ethics and scientific committee of the Hospital for Tropical Diseases in Ho Chi Minh City as a substudy of the Itraconazole Versus Amphotericin B for Penicilliosis (IVAP) randomized controlled trial (approval number 329/QD-BVBND). Control plasma samples were from a contemporaneous study of risk factors for *Streptococcus suis* infections (approval number 326/QD-BVBND). All participants gave informed consent for their specimens to be stored and used in this research.

### Study Design and Populations

In this retrospective diagnostic case-control study, cases included all patients with culture-proven talaromycosis who participated in the IVAP trial at 5 hospitals across Vietnam between 2012 and 2016. The IVAP trial recruited 440 of 573 (77.0%) talaromycosis patients who were screened for eligibility, representing a wide spectrum of disease severity [9]. The reference standard was culture-proven talaromycosis, defined as a compatible clinical syndrome plus positive cultures from blood and/or from skin lesions, lymph nodes, body fluids, or bone marrow aspirate. Blood culture was performed for all patients using the standard automated aerobic BACTEC bottles. Only plasma and urine specimens that were collected at the same time of the first blood culture collection were used for antigen testing. Control participants included healthy volunteers and patients who were diagnosed with any infectious diseases other than talaromycosis while hospitalized at the Hospital for Tropical Diseases between 2011 and 2017. HIV infection was excluded from the control group because a negative blood culture does not rule out talaromycosis in HIV-infected individuals [2].

### Analytical Validation of the Mp1p EIA

The recombinant Mp1p (rMp1p), mouse MAbs, and rabbit PAbs were obtained from Department of Microbiology, University of Hong Kong. The Mp1p EIA was validated in our laboratory at the Oxford University Clinical Research Unit in Vietnam. The analytical limit of detection (LoD) was determined using the 4-parameter logistic (4PL) curve fitting on 14 rMp1p concentrations ranging from 1 pg/mL to 10 000 pg/mL. The LoD was defined as the lowest rMp1p concentration corresponding to an optical density (OD) value, which was reliably distinguished from the mean OD of 3 blank samples [26]. A set of low (200 pg/mL), medium (800 pg/mL), and high (3200 pg/mL) rMp1p control concentrations was used for experiments to calculate intra-assay and interassay variability. The intra-assay variability was determined by running 10 replicates each of rMp1p controls in 1 experiment. The interassay variability was determined by running rMp1p controls in 10 separate experiments. The coefficient of variation (CV) for intra-assay variability should be <10%, and the CV for interassay variability should be <15% [27].

### Sample Preparation and Testing

The Mp1p EIA was performed on thawed plasma and urine samples. In brief, immunoplates (Nunc, Denmark) were coated with the rabbit Mp1p PAbs at a concentration of 5 μg/mL overnight at 4°C and were further blocked in Tris-base with 0.2% gelatin and 0.25% casein at 37°C for 2 hours. Aliquots of 100 μL of undiluted plasma and urine samples were added to the coated wells and incubated at 37°C for 1 hour. The plate was washed 6 times with phosphate-buffered saline with 0.05% Tween20 (Sigma, St Louis, Missouri). After washing, 100 μL of 1:1000 diluted mouse Mp1p MAbs conjugated with biotin was added and kept at room temperature (25°C) for 30 minutes, followed by incubation with streptavidin-horseradish peroxidase (Agilent-Dako, Santa Clara, California) for 30 minutes. Tetramethylbenzidine (Invitrogen, Carlsbad, California) was then added. The reaction was stopped after 10 minutes by the addition of 0.3 M sulfuric acid. The plate was then examined in an enzyme-linked immunosorbent assay reader (ACTGene, Piscataway, New Jersey) at 450 nm.

### Sample Size Estimates

Power calculations were based on the sensitivity and specificity of the Mp1p EIA we previously published [25], using the formula for estimating an infinite population proportion [28]. Assuming a sensitivity of 0.75 (error *d* = 0.05) and a specificity of 0.99 (error *d* = 0.01), a sample size of 289 cases and 381 controls would provide a power of 80% or higher to demonstrate a sensitivity of at least 70% and a specificity of at least 98%–100% (α = .05) for the Mp1p EIA.

### Statistical Analyses

The OD distribution between cases and controls were compared using Wilcoxon rank test. A receiver operating characteristic (ROC) curve was generated, which displayed all sensitivity and specificity pairs for different OD cutoff points using Prism 4.0 software. The assay cutoff was determined based on the Youden index on the ROC curve, which maximizes true positives and minimizes false positives. The discrimination power between cases and controls was determined by calculating the area under the ROC curve (AUC) and the 95% confidence intervals (CIs). The point estimates and the 95% CIs for sensitivity and specificity were calculated based on the reference standard. The sensitivities of the Mp1p EIA and of blood culture performed on the same patients were compared using McNemar test. Pairwise comparisons of the sensitivities of the Mp1p EIA performed in plasma, urine, and plasma plus urine from the same patients were performed using McNemar tests. All statistical analyses were performed using R software version 3.6.1.

## RESULTS

### Characteristics of Study Participants

**Figure 1.**
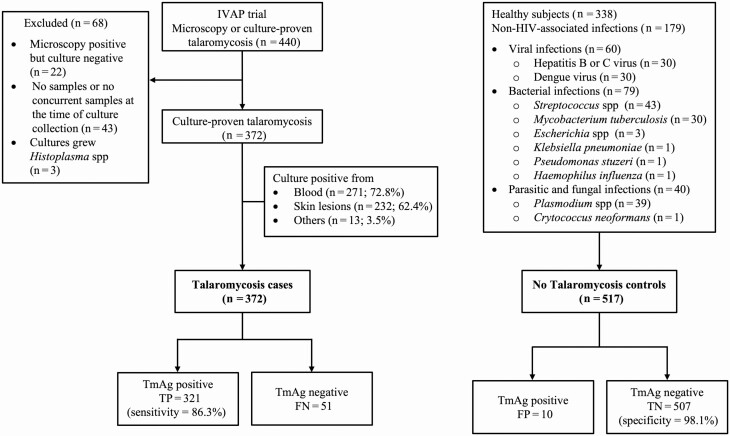
The flowchart describes the selection of the study population. Cases included 372 patients from the Itraconazole Versus Amphotericin for Penicilliosis trial who had culture-proven talaromycosis and who had plasma samples drawn at the same time of the first blood culture collection. Controls included 517 participants: 338 healthy volunteers and 179 human immunodeficiency virus–uninfected patients hospitalized with a range of common infections at the Hospital for Tropical Diseases in Ho Chi Minh City. Abbreviations: FN, false negative; FP, false positive; HIV, human immunodeficiency virus; IVAP, Itraconazole Versus Amphotericin for Penicilliosis; TmAg, *Talaromyces marneffei* antigen; TN, true negative; TP, true positive.

Figure 1 shows the selection of the study participants and the specific infectious disease diagnoses of the controls. Of the 440 patients who participated in the IVAP trial, 372 met the inclusion criteria. The median age was 33 (interquartile range [IQR], 29–38) years; 246 (66.1%) were male. All patients were living with HIV; the median CD4 count was 10 (IQR, 2–22) cells/μL. Blood culture was positive for Tm in 271 (72.8% [95% CI, 68.0%–77.2%]) patients. The control group included 517 participants: 338 healthy volunteers and 179 patients infected with a spectrum of bacterial, mycobacterial, viral, parasitic, and fungal pathogens. The median age among the controls was 37 (IQR, 27–48) years; 390 (75.4%) were male.

### Analytical Validation of the Mp1p EIA

Based on the standard curve of rMp1p concentrations and the corresponding OD values, the LoD was at least as low as 62.5 pg/mL, which was the same as the LoD generated in our laboratory in Hong Kong [25]. This demonstrates assay consistency between batches of antibodies and rMp1p and between laboratories. The intra-assay CV (for 10 replicates of 3 controls) was 1.4% (<10%), and the interassay CV (for 10 repeated experiments of 3 controls) was 9.2% (<15%), demonstrating good assay reproducibility.

### Diagnostic Accuracy of the Mp1p EIA in Plasma Samples

**Figure 2.**
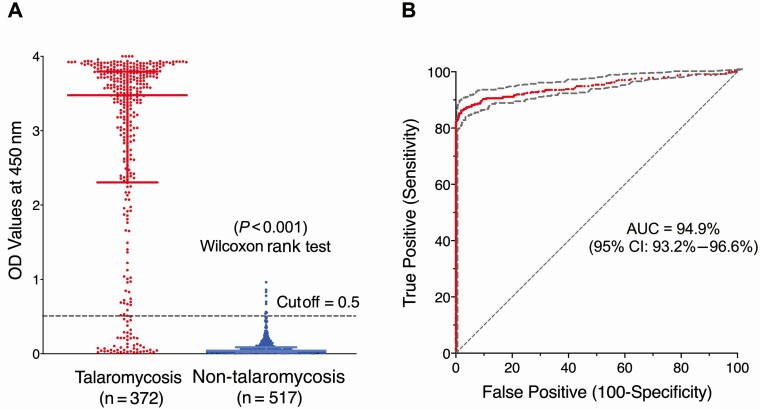
*A*, Optical density (OD) distribution of talaromycosis cases and non-talaromycosis controls; the difference in OD distribution was statistically significant. *B*, Receiver operating characteristic (ROC) curve demonstrated excellent discrimination (94.9% accuracy) between talaromycosis cases and non-talaromycosis controls. The OD cutoff of 0.5 was the Youden index calculated from the ROC curve, which maximizes true positives, minimizes false positives, and assumes equal importance of sensitivity and specificity. Abbreviations: AUC, area under the receiver operating characteristic curve; CI, confidence interval; OD, optical density.

Figure 2A displays the difference in OD distribution of cases and controls, which was statistically significant (*P *< .001, Wilcoxon rank test). Figure 2B shows the ROC curve plotting true positives (sensitivity) against false positives (1-specificity). The AUC demonstrated a 94.9% accuracy in the discrimination between participants with and those without talaromycosis.

At the OD cutoff of 0.5 generated by the Youden index, the diagnostic characteristics of the Mp1p EIA are shown in Table 1. The Mp1p EIA correctly diagnosed talaromycosis in 321 of 372 (86.3%) patients (95% CI, 82.3%–89.5%); this was superior to blood culture, which correctly diagnosed talaromycosis in 271 of 372 patients (72.8% [95% CI, 68.0%–77.2%]; *P *< .001, McNemar test). The Mp1p EIA correctly excluded talaromycosis in 507 of 517 (98.1%) patients (95% CI, 96.3%–99.0%). The positive likelihood ratio (LR) (where LR >1 indicates an association with disease) was 45. The negative LR (where LR <1 indicates an association with absence of disease) was 0.1.

**Table 1.** Clinical Performance of the Mp1p Enzyme Immunoassay in 889 Study Participants, Including 372 Participants With Culture-proven Talaromycosis and 517 Non-talaromycosis Control Participants

OD_cutoff_ = 0.5	Talaromycosis Cases	Non-talaromycosis Controls	Sum
TmAg positive OD ≥0.5	TP 321	FP 10	331
TmAg negative OD <0.5	FN 51	TN 507	558
Sum	372	517	889
Diagnostic Features	Diagnostic Values, No. (%)	(95% CI)	
Sensitivity	321/372 (86.3%)	(82.3%–89.5%)	
Specificity	507/517 (98.1%)	(96.3%–99.0%)	
Positive likelihood ratio	$\frac{0.863}{1-0.981}=45$		
Negative likelihood ratio	$\frac{1-0.863}{0.981}=0.14$		

Abbreviations: FN, false negative; FP, false positive; OD, optical density; TmAg, *Talaromyces marneffei* antigen; TN, true negative; TP, true positive.

### Sensitivities of Plasma Versus Urine in Paired Samples

Urine samples were collected in the IVAP trial, and paired plasma and urine samples were available for 269 of 372 (72.3%) talaromycosis cases. More talaromycosis patients were identified in urine than in plasma samples: 232 of 269 (86.2% [95% CI, 81.4%–90.0%]) vs 223 of 269 (82.9% [95% CI, 77.7%–87.1%]), but the difference did not reach statistical significance (*P* = .06, McNemar test). When testing plasma and urine in combination, 7 additional cases were identified, resulting in a significantly higher sensitivity (239/269; 88.8% [95% CI, 84.3%–92.2%]) compared to testing plasma alone (223/269; 82.9% [95% CI, 77.7%–87.1%]) (*P *< .001, McNemar test), or to testing urine alone (232/269; 86.2% [95% CI, 81.4%–90.0%]; *P* = .02, McNemar test).

### Characteristics of 10 Controls Who Tested Positive (False Positives) and 51 Cases Who Tested Negative (False Negatives)

The 10 false positives included 6 healthy volunteers and 4 patients who had 3 different infections. Their OD values were in the low positive range (0.54–0.96) and were not discriminatory between healthy volunteers and hospitalized controls. There was no clustering of infections to suggest cross-reactivity with a specific pathogen.

We compared the characteristics of the 51 false negatives and the 321 true positives to identify features associated with a false-negative test. The differences in age, sex, and proportion of patients who initiated antifungal therapy prior to enrollment were not statistically significant between the groups. The false negatives had fewer cases with positive blood culture (58.8% vs 74.1%; *P* = .036), and the time to positive culture was longer (9.0 vs 6.6 days; *P* = .003), indicating that lower blood fungal burden is associated with a false-negative test. Urine test was positive in 16 of 46 (34.8%) false-negative samples, suggesting that urine is more sensitive than plasma for Tm antigen testing. Urine samples were not available in control participants; therefore, assessment of specificity in urine could not be performed.

Incidentally, we discovered that 1 of the 5 study sites performs fungal blood cultures when a disseminated fungal infection is suspected. This is done using chloramphenicol-treated Sabouraud agar, and the cultures are kept for 10 instead of 5 days for the standard BACTEC blood culture system. Compared to the other 4 sites, this site has significantly higher proportion of patients with positive blood cultures (114/130 [87.7%] vs 154/242 [63.6%]; *P *< .001, χ ^2^ test), longer time to positive culture (7.6 ± 2.7 vs 6.2 ± 2.8 days; *P *< .001, *t* test), and therefore a lower sensitivity of the Mp1p EIA: 104 of 130 (80.0% [95% CI, 72.3%–86.0%]) vs 217 of 243 (89.7% [95% CI, 85.2%–92.9%]) (*P* = .01, χ ^2^ test).

## DISCUSSION

Relying on culture-based methods to diagnose talaromycosis is suboptimal. Blood cultures become positive only during late-stage infections, and the slow growth of the organism, taking up to 14 days to grow, contributes to the missed or late diagnosis of 30%–50% of infections [2, 10]. This has clear implications for the implementation of timely treatment, and indeed currently the diagnosis is made after death in 7% of patients [9]. While a presumptive diagnosis can be made rapidly based on microscopy of skin lesions, skin lesions are a late manifestation of disseminated disease and are absent in up to 60% of patients [10]. Delay in diagnosis is associated with high mortality [2, 3]. In our robustly powered study from Vietnam, we found the Mp1p EIA to have excellent clinical specificity (98.1% in 517 controls), consistent with our previous results from Hong Kong (99.4% in 540 controls) [25]. The sensitivity was superior to standard BACTEC blood culture (86.3% vs 72.8%), and higher than our previous estimate (75.0% in 20 cases), likely reflecting a more precise measure due to the increased sample size [25]. We also found the Mp1p EIA to be sensitive when testing urine, comparing favorably with plasma and offering significantly increased sensitivity when the result was paired with that of plasma testing, increasing from 82.9% to 88.8%. Furthermore, the time to result was substantially faster, permitting a diagnosis on average 6 days faster than would be confirmed by culture. Therefore, we believe our assay has the potential to significantly improve the management of patients with talaromycosis.

The Mp1p EIA offers several advantages over other MAb-based immunoassays in development. Comparing to the MAb-based 4D1 inhibitory EIA [20], the Mp1p EIA detects a Tm-specific mannoprotein [22, 25], rather than nonspecific cytoplasmic antigens. It directly measures antigen binding instead of competitive interference, and is simpler to perform. The sensitivity was reported to be 100% for the MAb-4D1 EIA [20] and was 87.9% for its point-of-care immunochromatographic platform [21]. However, similar to other antigen-detection studies for talaromycosis to date [14–18, 29], the sample sizes were small (n = 45 and n = 66, respectively). Reference samples were not systematically selected; all cases were blood culture positive, which is not representative of the disease spectrum in clinical practice, and thus is biased toward higher sensitivity. The prevalence of blood culture positivity in our study was 72.8% which is consistent with published real-world patient cohorts [2, 3, 30, 31]

An alternative approach for diagnosis to culture and antigen testing is nucleic acid amplification tests, such as real-time polymerase chain reaction (PCR). We found our assay to have higher sensitivity than real-time PCR assays in development (sensitivity range, 70%–80%) [32–36]. This is likely because the assay directly detects an antigen abundantly present in patients’ samples, whereas PCR assays require DNA extraction with substantial DNA loss. Moreover, the EIA is easier to perform, is more inexpensive, does not require sophisticated equipment or technician skills, and has the potential to be developed as a point-of-care test. We found that the Mp1p EIA produced consistent standard curves between laboratories and batches of antibodies, and was highly reproducible. These characteristics make it a good candidate for development for clinical use. A commercial version of the Mp1p EIA was approved in China in October 2018. Efforts to systematically validate the commercial Mp1p EIA and efforts to develop Mp1p point-of-care platforms are underway and are promising.

We made an incidental finding during the evaluation of false-negatives that has important implications for change of practice. We discovered that the use of a simple fungal blood culture method using an antibiotic-containing media and extending culture time from 5 to 10 days, as practiced at 1 of our study sites, resulted in a higher proportion of blood culture positivity (87.7% vs 63.6%). At this site, fungal blood cultures were performed at physician request only; the extension of this to all patients suspected of having invasive fungal infections might result in significantly increased microbiological confirmation.

Our study has limitations. First, we excluded PLWH from the control group because we could not exclude talaromycosis in PLWH on the basis of negative blood culture alone. Our specificity estimate is therefore not based on a population at high risk for talaromycosis. Second, our samples were collected between 2011 and 2017; protein in older samples may have significantly degraded, which is likely to lead to an underestimation of assay sensitivity. Finally, although this is the most robust diagnostic study for talaromycosis to date, the retrospective case-control design only gives estimates of diagnostic accuracy when the diagnosis is already known. Prospective diagnostic studies are required and are currently underway to determine the diagnostic utilities of the Mp1p EIA in patient populations who are at risk for talaromycosis (ClinicalTrials.gov identifier NCT04033120).

## CONCLUSIONS

The Mp1p EIA is highly specific and is superior to standard BACTEC blood culture in sensitivity and in time to result for diagnosing talaromycosis. Urine is as good as plasma as a substrate for antigen testing, and paired plasma and urine testing further improves sensitivity. Fungal blood culture may be superior to standard blood culture and should be performed for patients with advanced HIV suspected of having disseminated fungal infections.

## Notes

***Author*** ***contributions.*** Study concept and design: K. Y. Y. and T. L. Obtaining funding: T. L. Clinical and microbiology data acquisition: N. T. M. T., V. T. L., H. T. N., H. T. A. H., N. P. H. L., N. V. V. C., and T. L. Laboratory work: N. T. M. T., J. F. W. C., and J.-P. C. Data analyses: N. T. M. T., J. F. W. C., and T. L. Interpretation of data: N. T. M. T., J. F. W. C., P. C. Y. W., J. N. D., R. v. D., G. T., J. P., K. Y. Y., and T. L. Drafting the manuscript: N. T. M. T., J. F. W. C., K. Y. Y., and T. L. Critical revision of the manuscript for intellectual content: N. V. V. C., P. C. Y. W., J. N. D., R. v. D., G. T., and J. P. All authors contributed to and approved the final manuscript.

***Acknowledgments.*** The authors thank all patients who participated in this study; all investigators and study staff from 5 hospitals across Vietnam; and all supporting staff from the Clinical Trials Units at the Oxford University Clinical Research Unit in Vietnam who worked together over 5 years to deliver the IVAP trial.

***Financial support.*** This work was supported by the Medical Research Council, Department of International Development, and the Wellcome Trust (all in the United Kingdom) through the Joint Global Health Trials Grant (grant number G1100682); the National Institutes of Health (grant numbers R01AI143409 to T. L. and P30AI064518 with a Duke Center for AIDS Research’s Faculty Development subaward to T. L.); and donations of Marina Man-Wai Lee and the Hong Kong Hainan Commercial Association South China Microbiology Research Fund.

***Potential conflicts of interest.*** P. C. Y. W. has provided scientific advisory/laboratory services for Gilead Sciences, International Health Management Associates, Merck & Co, and Pfizer. J. P. reports research grants from Merck, Astellas, F2G, Scynexis, Pfizer, Amplyx, Ampili, Minnetronix, and Matinas; and nonfinancial support from Merck, Astellas, F2G, and Scynexis, outside the submitted work. K. Y. Y. has been issued US Patent 5973131. T. L. has received investigator-initiated research funding from Gilead Sciences, outside the submitted work. All other authors report no potential conflicts of interest. All authors have submitted the ICMJE Form for Disclosure of Potential Conflicts of Interest. Conflicts that the editors consider relevant to the content of the manuscript have been disclosed.
